# Dentition and Its Derangements

**Published:** 1863-02

**Authors:** A. Jacobi


### Selections.

Dentition and its Derangements—*By A. Jacobi*, M. D. *LECTURE XII.*—Part 1.—The remarks made in my last lecture on the various causes of convulsions, and the influence of some irritation of the dental nerves in bringing them on, enable us to classify their etiology in the following manner. Convulsions result from

I. Direct irritation either
   1. in the peripheric course of motory nerves, or
   2. in their origin in their central organ.

II. Irritation of sensitive nerves, the grey substance of the nervous centres being the joining link between the sensitive and the motory nerves,
   1. consciousness being entirely excluded—reflex—convulsions proper.
   2. consciousness being more or less excluded; the brain is the proximate seat.

III. Anomalous nutrition of the nervous tissue
   1. in the peripheric nerves,
   2. in the brain,
   3. in the spine.

In this latter class the morbid symptoms are either permament, or temporarily induced by either
   1. direct irritation, or
   2. irritation of and through the sensitive nerves, or
   3. voluntary action.

In this classification we cannot include the sometimes permanent contraction of the muscles, which does not at all depend on any morbid condition, or irritation, of the nervous system or a single nerve; nor those which, it is true, are brought on by eitheir direct or reflected irritation, or by voluntary action, but at all events require a morbid condition of the muscular substance.

Although the above classification is thoroughly physiological and rational, you perceive at once the difficulty of bringing every case of convulsions occurring in practice, under its proper head. It is entirely impossible to know enough of the history of the patient and of the immediately preceding irritations in every given case, to determine its character. But this much is easily understood, from what I have said, that the large majority of cases of convulsions are of but a symptomatic character, and further, that the proximate causes may be very various and numerous. Therefore it follows, that the practice of explaining attacks of convulsions occurring during dentition, that is, a period of life where a large number of unwonted influences are brought to bear upon the unresisting infantile organism, by nothing but the irritation of the dental ramifications of the fifth pair of cerebral nerves—is entirely one-sided and unjustified. It is true, however, that the distinction between the various possible causes requires more pathological knowledge and diagnostic ability than mere dictatorial airs and the common practice of wantonly cutting down into the gums of a child affected with some unexplained ailment. Always be aware that convulsions are no disease, but the symptom of some morbid alteration or irritation, and that these alterations and irritations are very numerous and various, and that of those very numerous and various irritations, the protrusion of a tooth under more or less unfavorable circumstances, may be one.

Now, after you have reviewed the principal points of the etiology of convulsions, and found out with me the possible connection of dentition and convulsions, it remains to speak of the connection of the same process with paralysis, as occurring in young children. Most, but by far not all, of the cases of paralysis in infantile are observed at the period of dentition, that is, about or after the middle of the first year. Inasmuch as the age in which this affection was principally observed, struck the minds of observers most, it was called *infantile* paralysis; because its etiology and nature were not understood, it was baptized *essential*, as we are pleased to name everything the nature of which we do not know, essential or specific; and as it was generally observed at the time of dentition, and thought to depend on some difficulties in the protrusion of teeth, it was called *dental*. Thus the terms essential, infantile, dental paralysis, so common in both practice and medical literature, are synonymous.

Infantile paralysis, although not a common, is not an unfrequent disease, and an important one both for its practical bearings and its scientific interest. I shall try to explain, as far as possible, its etiology and nature; but before that I

shall have to review the principal views held by the leading authors on our subject. Among the first ranks Dr. Von Heine, with book on *Spinal Infantile Paralysis*, who, in the attempt to provide an anatomical foundation for the peculiar symptoms of the disease, takes all the genuine cases of infantile paralysis for spinal affections. But the description of the characteristics of the disease, as found in his work, fully coincides with the teachings of other authors, and with what observations every one of you will have to make in his own future practice.

Essential, dental, or infantile paralysis, runs its course in two distinct stages the first of which is very short, so short, indeed, sometimes, that it is not at all or hardly noticed. An infant exhibits some slight symptoms of fever in the evening, is put to bed, sleeps more or less, sometimes quite well, and is paralysed when taken up. Sometimes, however, the initiatory symptoms are more serious, high fever, congestion, cerebral irritation, symptoms of meningitis, or of "difficult dentition" being present. Patient is restless, screams in paroxysms, the eyes are half open in the interrupted sleep. Vomiting, diarrhœa, or the symptoms of rheumatic fever are sometimes observed. Or the first symptoms of an eruptive fever, with or without convulsions, or an attack of convulsions depending on one or more of the above mentioned causes, isolated or repeated, will be observed. The child becomes tired, quiet, and is paralysed. Generally the lower extremities are affected, sometimes an upper one at the same time; frequently a single lower extremity, without an affection of the arms; in some cases paralysis is so local as to affect single muscles only. The urinary bladder and rectum are sometimes debilitated, but never paralysed for a longer period. The whole number of paralytic cases recorded by Dr. Heine amounts to 192. Of these 158 were such as he comprehends under the head of spinal infantile paralysis. Of these were—

| | Males. | Females. | Total. |
|---|---|---|---|
| Paraplegia | 17 | 20 | 37 |
| Hemiplegia | 18 | 16 | 34 |
| Partial paralysis | 44 | 40 | 84 |

Paralysis of one arm was observed in two cases; it was never cured, and was never complicated with paralysis of any other organ. Paralytic lordosis was observed in a single case.

The second stage, that is, fully developed paralysis, is as long as the former is short. Vital turgor is diminished, skin and muscles are flabby. The sensitive nerves are not at all or but little affected; where there is in the begining paralysis of the trunk and arm it disappears, with the exception of a weakness of the muscles of the back sufficient to give rise to paralytic scoliosis. Where the two lower extremities are affected, one will gradually recover its mobility, and sometimes nothing will remain of the original disease but the paralysis of some muscles, principally the peronæi. There is, however, seldom a thorough improvement after the first four or eight weeks; then the temperature sinks, feet and muscles become atrophic, the bones decrease in both length and thickness. The muscles will shorten, the tendo Achillis being the first to contract; in consequence of repeated attempts at locomotion, deformities will at last show themselves. Lateral curvatures of the vertebral column are very frequent: a bluish tint of the skin, frostbites, and ulcerations are the results of the diminished efforts of the heart and arteries. Bowels operate slowly and insufficiently in many cases; menstruation is not interfered with, and in a single case was even observed at the early age of twelve years. Neither mind nor senses are affected. The renal secretion is more or less normal; some have found a superabundance of phosphate of lime at the time of the commencing atrophy of the muscles, but not all the authors have obtained the same result. The diseases peculiar to early age do not prove particularly dangerous, so that some patients have been known to reach an advanced age. Hutin records the case of a man who died at forty-nine years.

A year or two after the appearance of paralysis, deformities begin to develop themselves, particularly in the lower extremities. Their principal forms are the paralytic varieties of talipes varus, valgus, equinus, calcaneus, contractions of the hip and the knee-joint, retrocurved, inverted, and everted knee. They depend on the want of equality as far as the degree of muscular paralysis is concerned; the completely paralysed muscles having lost their power, while their antagonists are still active to a certain degree. Thus talipes equinus is the result of contraction of the tendo Achillis which is not counteracted by an antagonist; the same affection combined with paralysis of the peronæi muscles produces talipes varus; paralysis of the anterior and posterior tibial muscles: talipes valgus; paralysis of tendo Achillis: talipes calcanens. In a similar manner, in hemiplegia and paraplegia, where the extensors of the legs are paralysed, contraction of the knee is brought on; and adduction of the thigh to the pelvis is the result of paralysis of the extensors of the thigh. Of the mentioned deformities t. equinus comes usually first, t. varus next, t. valgus third, and lastly t. calcaneus. The differential diagnosis between this affection from cerebral and spasmodic hemiplegia is simple and easy; for in this latter muscular retraction sets in with the acute cerebral affection. Paralysis of the arm generally gives no rise to contraction and deformity, as mostly both flexors and extensors are equally affected, and no antogonistic muscular action is called forward by any disturbance of the equilibrium.

The etiology of infantile paralysis appears simple enough, if we are to believe the assertions of Dr. Von Heine. The nervous centres undergo a rapid physiological development during the first years of life; in this period infantile paralysis is principally observed. Therefore there is a great liability to alterations of the brain and spine, in grave cases of difficult dentition, or severe exanthematic fevers, in consequence of hyperæmia, congestion, and irritation. The usual results of the processes are cerebral and spinal meningitis, with serious or bloody transudations, followed by pressure and paralysis. The age of such children is a very predisposing element, as about this period of life those pathological changes are of common occurrence; a further predisposition is attributed by the author to the very healthy, robust, and vigorous constitution of those generally affected, also to the presence of general symptoms of fever (also in other diseases), high temperature, congestion, convulsions, fright, screaming, and vertebral pain in such children as are sufficiently advanced to explain their sensations. Thus the author sees the cause of infantile paralysis in spinal effusions, in some perhaps in extravasations. The former suposition he considers as founded on the fact, that, (by resorption, he says,) a complete paralysis of the trunk and extremities will very generally disappear, to a certain extent, during the first period of the whole process. As to the results obtained by post-mortem examinations, they are very few indeed; for as infantile paralysis has in itself no tendency to terminate fatally, there are very few examinations on record. At all events, however, there are few which by their pathological remnants proved the spinal origin of the cases. As for other results found in the dead, they give no clue to the anatomical cause of the affection. Such, for instance, is the atrophy of the limbs, especially the muscles, and the degeneration of the latter into adipose, and in one instance even cellular tissue. Nerves and arteries require, it is true, a longer time, and have less tendency to become atrophied and degenerate, but they have been found to be so.

According to Dr. Heine, we need not wonder at the frequency of spinal diseases of local character. It is sufficiently explained by its normal hyperæmia, and its functional character. Lesions of the entire spine, however, are very rare; but Prof. Schiff has proved by experiments, that the affection of a limited part of the medullary substance of the spine may result in complete paralysis. Generally a leison ef the right side will be followed by paralysis on the right side, and *vice versa*. The action of the sensitive nerves may remain normal, a circular pain only being felt in cases of mere compression of the spine, depending on dilatation of the blood-vessels, and effusion, or certain other diseases of the membranes. This circular pain even may be absent when the anterior lateral parts of the spine are affected. In cases of very superficial and partial affections of the spine, paralysis will be partial in a corresponding degree.

